# Vindoline Attenuates Osteoarthritis Progression Through Suppressing the NF-κB and ERK Pathways in Both Chondrocytes and Subchondral Osteoclasts

**DOI:** 10.3389/fphar.2021.764598

**Published:** 2022-01-12

**Authors:** Meisong Zhu, Qiang Xu, Xinmin Yang, Haibo Zhan, Bin Zhang, Xuqiang Liu, Min Dai

**Affiliations:** Department of Orthopedics, The First Affiliated Hospital of Nanchang University, Artificial Joints Engineering and Technology Research Center of Jiangxi Province, Nanchang, China

**Keywords:** vindoline, osteoarthritis, extracellular matrix, osteoclastogenesis, NF-κB pathway, ERK pathway

## Abstract

Disruption of extracellular matrix (ECM) homeostasis and subchondral bone remodeling play significant roles in osteoarthritis (OA) pathogenesis. Vindoline (Vin), an indole alkaloid extracted from the medicinal plant *Catharanthus roseus*, possesses anti-inflammatory properties. According to previous studies, inflammation is closely associated with osteoclast differentiation and the disorders of the homeostasis between ECM. Although Vin has demonstrated effective anti-inflammatory properties, its effects on the progression of OA remain unclear. We hypothesized that Vin may suppress the progress of OA by suppressing osteoclastogenesis and stabilizing ECM of articular cartilage. Therefore, we investigated the effects and molecular mechanisms of Vin as a treatment for OA *in vitro* and *in vivo*. In the present study, we found that Vin significantly suppressed RANKL-induced osteoclast formation and obviously stabilized the disorders of the ECM homeostasis stimulated by IL-1β in a dose-dependent manner. The mRNA expressions of osteoclast-specific genes were inhibited by Vin treatment. Vin also suppressed IL-1β-induced mRNA expressions of catabolism and protected the mRNA expressions of anabolism. Moreover, Vin notably inhibited the activation of RANKL-induced and IL-1β-induced NF-κB and ERK pathways. *In vivo*, Vin played a protective role by inhibiting osteoclast formation and stabilizing cartilage ECM in destabilization of the medial meniscus (DMM)-induced OA mice. Collectively, our observations provide a molecular-level basis for Vin’s potential in the treatment of OA.

## Introduction

Osteoarthritis (OA) is a common and disabling joint disease in the elderly. It is characterized by progressive cartilage deterioration, destruction of subchondral bone, pain, joint rigidity and eventually dysfunction, seriously affecting the life quality of patients, (Hunter and Bierma-Zeinstra, 2019; Shao et al., 2020), and causing enormous economic and medical burden to society. (Hunter et al., 2014). Traditionally, the treatment for OA includes pain management with joint replacement for end-stage disease, (Glyn-Jones et al., 2015), but it is insufficient to slow, stop, or reverse the joint damage. (Zhang et al., 2016). In addition, prosthesis lifespan remains a major problem. (Glyn-Jones et al., 2015). Therefore, there is an urgent need to explore safe and effective treatments for OA.

To date, the pathophysiology of OA remains unclear. (Zheng et al., 2021). The disorders of the homeostasis between extracellular matrix (ECM) synthesis and degradation are indispensable for the onset of OA. (Shi et al., 2019). The dense ECM, mainly composed of type II collagen (COL2a1) and aggrecan, (Luo et al., 2017), plays an important role in the biomechanical properties of cartilage. When the joints move, ECM acts as elastic support to disperse the pressure and shear force. (Shi et al., 2019). However, there are many inflammatory factors in the OA joint. Among them, interleukin-1 beta (IL-1β), a proinflammatory cytokine considered as a major player in OA, could inhibit anabolic activities of chondrocytes thereby downregulating the synthesis of ECM components. In addition, IL-1β could stimulate chondrocytes to release several proteolytic enzymes, among which are the matrix metalloproteinases (MMPs). (Kapoor et al., 2011). Among the MMPs, MMP13 is a substrate-specific enzyme for COL2a1 degradation, and it could also degrade other proteins in articular cartilage, such as aggrecan, gelatin, osteonectin, and perlecan. (Xia et al., 2014). Therefore, inhibition of IL-1β and IL-1β-induced proteolytic enzymes to stabilize ECM of articular cartilage has been considered as a therapy strategy to protect against osteoarthritis, which has been proven by many studies. (Wang et al., 2017; Shi et al., 2019; Guo et al., 2021).

Increasing evidence has revealed that subchondral bone remodeling plays a key role in OA. (Siebelt et al., 2014; Zhu et al., 2020). Normal subchondral bone could provide both mechanical and nutritional supports for cartilage. In early-stage OA, subchondral bone remodeling, characterized by increased bone resorption and microstructure changes, adversely affects the biomechanical environment of the overlying cartilage. (Li et al., 2014; Zhu et al., 2019; Zhu et al., 2020; Hu et al., 2021). As unique bone-resorbing cells, osteoclasts are the major contributors to subchondral bone remodeling. Therefore, it has become a new target for the treatment of OA through inhibition of the osteoclast differentiation and activation to decrease subchondral bone remodeling. (Jiang et al., 2018; Zhou et al., 2019; Shao et al., 2020).

Natural compounds extracted from traditional herbal plants have attracted increasing attention for the treatment of OA. (Cameron and Chrubasik, 2014). Vindoline (Vin), an indole alkaloid extracted from the medicinal plant *Catharanthus roseus*, possesses antioxidant, anti-inflammatory, and anti-hypertriglyceredemia properties. (Goboza et al., 2019). Moreover, previous studies have shown that Vin suppresses receptor activator of nuclear factor kappa B (NF-κB) ligand (RANKL)-induced osteoclastogenesis and ovariectomy-induced osteoporosis in mice. (Zhan et al., 2020). Although Vin has demonstrated effective anti-osteoclastogenesis and anti-inflammatory properties, its effects on the progression of OA remains to be investigated. We hypothesized that Vin could act as a new means to treat OA *via* stabilizing ECM of articular cartilage and suppressing osteoclastogenesis. Accordingly, we investigated the effects and molecular mechanisms of Vin for OA treatment *in vitro* and *in vivo*.

## Material and Methods

### Chemicals, Media, and Reagents

**FIGURE 1:**
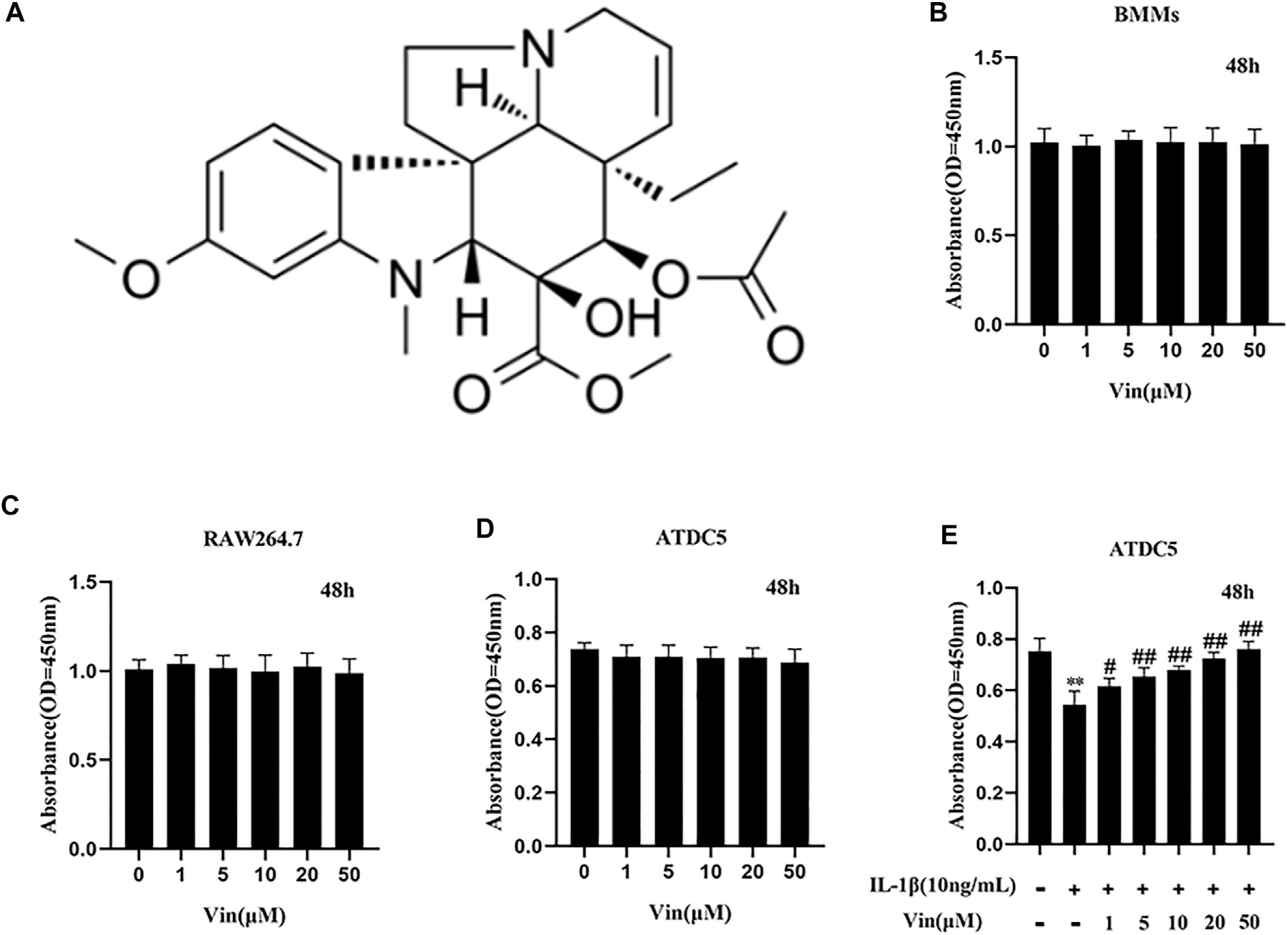
The chemical structure of Vin and results of the cell viability assays **(A)** The chemical structure of Vin. **(B–D)** Cell viability of bone marrow macrophages (BMMs), RAW264.7 and ATDC5 cells after treated with different concentrations of Vin for 48 h **(E)** ATDC5 cells were co-treated with IL-1β (10 ng/ml) and various concentrations of Vin for 48 h, and the cell viability was detected by CCK-8 assay. (*p < 0.05; **p < 0.01 vs the control group; #*p* < 0.05; ##*p* < 0.01 vs. the IL-1β-treated group).

Vindoline (Vin), 99.33% purity, was purchased from MedChem Express (Monmouth, NJ, USA) and the molecular structure is presented in Figure 1A. Dimethyl sulfoxide was used to dissolve Vin to yield a stock solution (50 mM), which was stored at −80°C. Dulbecco’s modified Eagle’s medium (DMEM)-high glucose was bought from Solarbio (Beijing, China). Fetal bovine serum (FBS) and DMEM/F12 were from Gibco (Grand Island, NY, United States). Cell Counting Kit-8 (CCK-8) and recombinant mouse RANKL were from BestBio (Shanghai, China) and R&D Systems (Minneapolis, MN, United States), respectively. Recombinant human macrophage colony-stimulating factor (M-CSF) and recombinant murine IL-1β were obtained from PeproTech (Rocky Hill, NJ, USA). The staining kit for tartrate-resistant acid phosphatase (TRAP) was from Sigma-Aldrich (St. Louis, MO, United States). Toluidine blue solution was purchased from Solarbio (Beijing, China). Primary antibodies against extracellular signal–regulated kinase (ERK), inhibitor of NF-κB (IκBα), p-65, phosphorylated-ERK (p-ERK; Thr202/Tyr204), p-p65 (Ser536), nuclear factor of activated T cells 1 (NFATc1), c-fos and glyceraldehyde-3-phosphate dehydrogenase (GAPDH) were from Cell Signaling Technology (Danvers, MA, United States). Primary antibodies against COL2a1, aggrecan and MMP13 were obtained from Boster Biological Technology (Wuhan, China).

### Cell and Cell Culture

The chondrogenic ATDC5 cell line was purchased from Riken Cell Bank (Ibaraki, Japan) and maintained in DMEM/F12 containing FBS (10%), and penicillin/streptomycin (1%). Before the following experimental treatment, ATDC5 cells were incubated for 2 weeks in the presence of ITS (10 μg/ml insulin, 5.5 μg/ml transferrin, and 6.7 ng/ml sodium selenite; Invitrogen) to induce chondrocytic differentiation. (Wang et al., 2020). The RAW264.7 murine macrophage cell line was obtained from American Type Culture Collection (Rockville, MD, United States) and cultured in DMEM containing FBS (10%) and penicillin/streptomycin (1%). Primary bone marrow macrophage cells (BMMs) were collected from the bone marrow of C57BL/6 mice (4–6 weeks old). Briefly, cells were flushed from the femur bone marrow with DMEM containing M-CSF (30 ng/ml), FBS (10%), and penicillin/streptomycin (1%), and cultured in a T75 flask for 24 h. Non-adherent cells were then removed, and the adherent cells were cultured for a further 3–4 days until cells were fully confluent. (Liu et al., 2014). Before the following experiments, all the cells were maintained under standard adherent conditions at 37°C under 5% CO_2_ and humidified atmosphere.

### Cell Viability Assay

The effect of Vin on cell viability was assessed using the CCK-8 assay kit. Briefly, BMMs were seeded in 96-well plates at a density of 1 × 10^4^ cells per well in triplicate. After 24 h of incubation, BMMs were treated with different concentrations of Vin (0–50 μM) for 48 h. Next, 10 μL CCK-8 solution was added to each well, and the cells were incubated at 37°C under 5% CO_2_ and humidified atmosphere for 2 h. Then, the absorbance at 450 nm was measured using an ELX800 microplate reader (Bio-Tek Instruments, Inc., Winooski, VT, United States). Similarly, the cytotoxic effects of Vin were also tested on the RAW264.7 and ATDC5 cell lines using the CCK-8 assay. In addition, ATDC5 cells were co-treated with IL-1β (10 ng/ml) and various concentrations of Vin (0–50 μM) for 48 h, and the cell viability was measured by CCK-8 assay.

### Osteoclast Differentiation Assay

BMMs were seeded in 96-well plates at a density of 1 × 10^4^ cells per well in triplicate and cultured in DMEM supplemented with M-CSF (30 ng/ml), RANKL (50 ng/ml), FBS (10%), penicillin/streptomycin (1%) and different concentrations of Vin (0, 5, 10, and 20 μM). The culture medium was replaced every 2 days for 5–7 days. Then the cells were fixed with 4% paraformaldehyde for 30 min and stained using the TRAP kit. The number and area of TRAP-positive cells (≥3 nuclei) were quantified.

### High Density Culture for ATDC5 and Toluidine Blue Staining

ATDC5 cells were cultured by high density culture method as mentioned in the previous study with some modifications. (Greco et al., 2011). Briefly, a total of 10 μl of the ATDC5 cell suspension was seeded in 24-well plates at a density of 1.5 × 10^7^ cells per well in triplicate. After 1 h of incubation, the cells adhered to the wall, and 500 μl DMEM/F12 containing FBS (10%), and penicillin/streptomycin (1%) was added into per well to culture ATDC5 cells for 24 h. Next, IL-1β (10 ng/ml) and various concentrations of Vin (0, 5, 10, and 20 μM) were added. The culture medium was changed every 2 days. After culturing the cells for 7–9 days, we fixed the cells in 4% paraformaldehyde for 30 min and stained them using the toluidine blue staining. The average optical density was calculated by ImageJ software (National Institutes of Health, Bethesda, MD, United States) to reveal the intensity of staining.

### RNA Extraction and Quantitative Real-Time Polymerase Chain Reaction (qPCR) Analysis

BMMs were seeded in 6-well plates at a density of 1 × 10^5^ cells/well and stimulated with RANKL (50 ng/ml) and M-CSF (30 ng/ml) in the presence or absence of Vin (10 μM) for 5–7 days. ATDC5 cells were also seeded in 6-well plates at a density of 2 × 10^5^ cells/well and stimulated with IL-1β (10 ng/ml) with or without Vin (10 μM) for 48 h. Total RNA was extracted from the cells using TRIzol (TransGen Biotechnology Co., Beijing, China) according to the manufacturer’s protocol. RNA (1 µg) was reverse-transcribed to complementary DNA using reverse transcriptase (TaKaRa Bio, Otsu, Japan) in accordance with the protocol of the manufacturer. SYBR Premix Ex Taq kit (TaKaRa Bio) was used for real-time PCR using the ABI StepOnePlus System (Applied Biosystems, Foster City, CA, United States). The thermocycling conditions of qPCR were as follows: denaturation at 95°C for 5 s, followed by amplification at 60°C for 30 s, for 40 cycles. GAPDH was used as housekeeping gene and all reactions were performed in triplicate. The specific primers of GAPDH, c-fos, NFATc1, TRAP, dendritic cell-specific transmembrane protein (DC-STAMP), calcitonin receptor, vacuolar-type ATPase d2 (V-ATPase d2), COL2a1, sex-determining region Y box 9 (SOX9), aggrecan, MMP13, a disintegrin and metalloproteinase with thrombospondin motifs 4 (ADAMTS4) and ADAMTS5 are shown in Table 1.

**TABLE 1:** Primer sequences for q-PCR.

Genes	Primer sequences
GAPDH	Forward 5ʹ-ACC​CAG​AAG​ACT​GTG​GAT​GG-3′
	Reverse 5ʹ-CAC​ATT​GGG​GGT​AGG​AAC​AC-3′
c-fos	Forward 5ʹ-CCA​GTC​AAG​AGC​ATC​AGC​AA-3′
	Reverse 5ʹ-AAG​TAG​TGC​AGC​CCG​GAG​TA-3′
NFATc1	Forward 5ʹ-GAG​TAC​ACC​TTC​CAG​CAC​CTT-3′
	Reverse 5ʹ-TAT​GAT​GTC​GGG​GAA​AGA​GA-3′
TRAP	Forward 5ʹ-TCA​TGG​GTG​GTG​CTG​CT-3′
	Reverse 5ʹ-GCC​CAC​AGC​CAC​AAA​TCT-3′
DC-STAMP	Forward 5ʹ-AAA​ACC​CTT​GGG​CTG​TTC​TT-3
	Reverse 5ʹ-AAT​CAT​GGA​CGA​CTC​CTT​GG-3
Calcitonin receptor	Forward 5ʹ-TGC​AGA​CAA​CTC​TTG​GTT​GG-3ʹ
	Reverse 5ʹ-TCG​GTT​TCT​TCT​CCT​CTG​GA-3ʹ
V-ATPase d2	Forward 5ʹ-AAG​CCT​TTG​TTT​GAC​GCT​GT-3′
	Reverse 5ʹ-TTC​GAT​GCC​TCT​GTG​AGA​TG-3′
COL2a1	Forward 5ʹ-GCC​AGG​ATG​CCC​GAA​AAT​TAG-3ʹ
	Reverse 5ʹ-ACG​ATC​ACC​TCT​GGG​TCC​TT-3ʹ
SOX9	Forward 5ʹ-GTG​CAA​GCT​GGC​AAA​GTT​GA-3′
	Reverse 5ʹ-TGC​TCA​GTT​CAC​CGA​TGT​CC-3′
Aggrecan	Forward 5ʹ-AGG​ATG​GCT​TCC​ACC​AGT​GC-3ʹ
	Reverse 5ʹ-TGC​GTA​AAA​GAC​CTC​ACC​CTC​C-3ʹ
MMP13	Forward 5ʹ-GAC​CCC​AAC​CCT​AAG​CAT​CC-3′
	Reverse 5ʹ-CCT​CGG​AGA​CTG​GTA​ATG​GC-3′
ADAMTS4	Forward 5ʹ-ATG​GCC​TCA​ATC​CAT​CCC​AG-3ʹ
	Reverse 5ʹ-GCA​AGC​AGG​GTT​GGA​ATC​TTT​G-3ʹ
ADAMTS5	Forward 5ʹ-GGA​GCG​AGG​CCA​TTT​ACA​AC-3′
	Reverse 5ʹ-CGT​AGA​CAA​GGT​AGC​CCA​CTT​T-3′

### Western Blot Analysis

RAW264.7 and ATDC5 cells were seeded in 6-well plates after pretreated with or without Vin (20 μM). Two hours later, RAW264.7 cells were stimulated with 0 or 50 ng/ml RANKL for 10 min and 48 h, and ATDC5 cells were treated with 0 or IL-1β (10 ng/ml) for 30 min and 48 h. Subsequently, total proteins were harvested from treated RAW264.7 and ATDC5 cells using radioimmunoprecipitation assay (RIPA) lysis buffer (Applygen Technologies Inc., Beijing, China) containing protease and phosphatase inhibitor supplementation (Sigma-Aldrich, Rockford, United States). Lysates were centrifuged at 12,000 × g for 10 min, and the supernatants were collected. The protein concentration was quantified using the bicinchoninic acid assay.

Protein samples (20 μg/well) were separated by sodium dodecyl sulfate–polyacrylamide gel and transferred onto polyvinylidene fluoride membranes (0.45 μm, Millipore, Bedford, MA, United States). Membranes were blocked by incubation in bovine serum albumin or 5% non-fat milk for 2 h at room temperature, and then incubated with primary antibodies overnight at 4°C. Subsequently, these membranes were washed with Tris-buffered saline-Tween 20 and incubated with the corresponding secondary antibodies for 1 h at room temperature. The protein bands were detected using Odyssey V3.0 image scanner (LiCOR Biosciences, Lincoln, NE, United States) and analyzed by ImageJ software. GAPDH was used as an internal control.

### Immunofluorescence Staining of p65

After treatment, 4% paraformaldehyde was used to fix the cells for 15 min followed by permeabilization with 0.5% Triton X-100 for 20 min. Then the cells were blocked by incubation in bovine serum albumin for 30 min at room temperature. Subsequently, the cells were incubated with anti-p65 antibody (1:400) overnight at 4°C, followed by incubation with the corresponding secondary antibody for 1 h at room temperature. Finally, 4, 6-diamidino-2-phenylindole (DAPI) was used to stain the nuclei for 5 min, and the stained cells were observed by LSM5 confocal microscope (Carl Zeiss, Oberkochen, Germany).

### DMM-Induced OA Mouse Model

All animal experiment procedures were performed in accordance with the Animal Ethics Committee of Nanchang University. OA model was established by destabilization of the medial meniscus (DMM) as previously mentioned. (Glasson et al., 2007). Briefly, C57BL/6 mice (8 weeks old, *n* = 18) were anesthetized with peritoneal injection of 10% chloral hydrate. A microsurgical knife was used to transect the attachment of the medial meniscus to the tibial plateau of the right knee (medial meniscotibial ligament). The lateral meniscotibial ligament should be carefully identified and protected during the surgery. All animals were assigned randomly to three groups: non-DMM control (sham group), DMM group, and DMM with 5 mg/kg Vin (Vin group). In sham group, only the right knee arthrotomy was performed without the transaction of medial meniscotibial ligament. In Vin group, mice received intraperitoneal injections of 5 mg/kg Vin once every 2 days for 8 weeks. In sham and DMM groups, mice were given the same amount of phosphate-buffered saline (PBS). Ultimately, all mice were sacrificed at 8 weeks post-surgery, and the samples of knee joint tissues were collected for further evaluation.

### Histological Assessment

Knee joint samples were collected and fixed in 4% paraformaldehyde for 48 h, followed by decalcification with 10% ethylenediaminetetraacetic acid (EDTA) for 2 weeks. Then the specimens were embedded in paraffin and performed by sagittal section. Subsequently, the histological sections were stained with Safranin O-Fast Green staining and TRAP staining. Osteoarthritis Research Society International (OARSI) scoring system was calculated to assess the destruction of joint cartilage. (Glasson et al., 2010). In addition, the histological sections were subjected to immunofluorescence (p-p65, p-ERK and aggrecan) and immunohistochemical (MMP13) staining, and heart, liver, spleen, lung, kidney organs were subjected to haematoxylin and eosin (HE) staining.

### Statistical Analysis

All experiments were performed independently at least three times, and the quantitative data are presented as the mean ± standard deviation (SD). Statistical analyses were performed using IBM SPSS Statistics v23.0 software (IBM Corp., Armonk, NJ, United States). Statistical significance was assessed using the Student t-test and one-way ANOVA. A *p* value < 0.05 was considered statistically significant.

## Results

### Effects of Vin on BMMs, RAW264.7 and ATDC5 Cells Viability

The potential cytotoxicity of Vin on precursor of osteoclasts (BMMs and RAW264.7 cells) was assessed by CCK-8 assay. Our results showed that Vin displayed no cytotoxicity on BMMs and RAW264.7 at the concentrations of 0–50 µM **(** Figures 1B,C). Then we assessed the effects of Vin on ATDC5 cells with or without IL-1β-stimulated inflammatory condition. We found that Vin also had no effect on ATDC5 cell viability at the concentrations of 0–50 µM (Figure 1D). In addition, Vin could significantly prevent the suppressive effects of IL-1β stimulation on cell viability dose-dependently in ATDC5 cells (Figure 1E).

### Vin Inhibits Osteoclastogenesis Induced by RANKL and Stabilizes the Disorders of the ECM Homeostasis Stimulated by IL-1β

**FIGURE 2:**
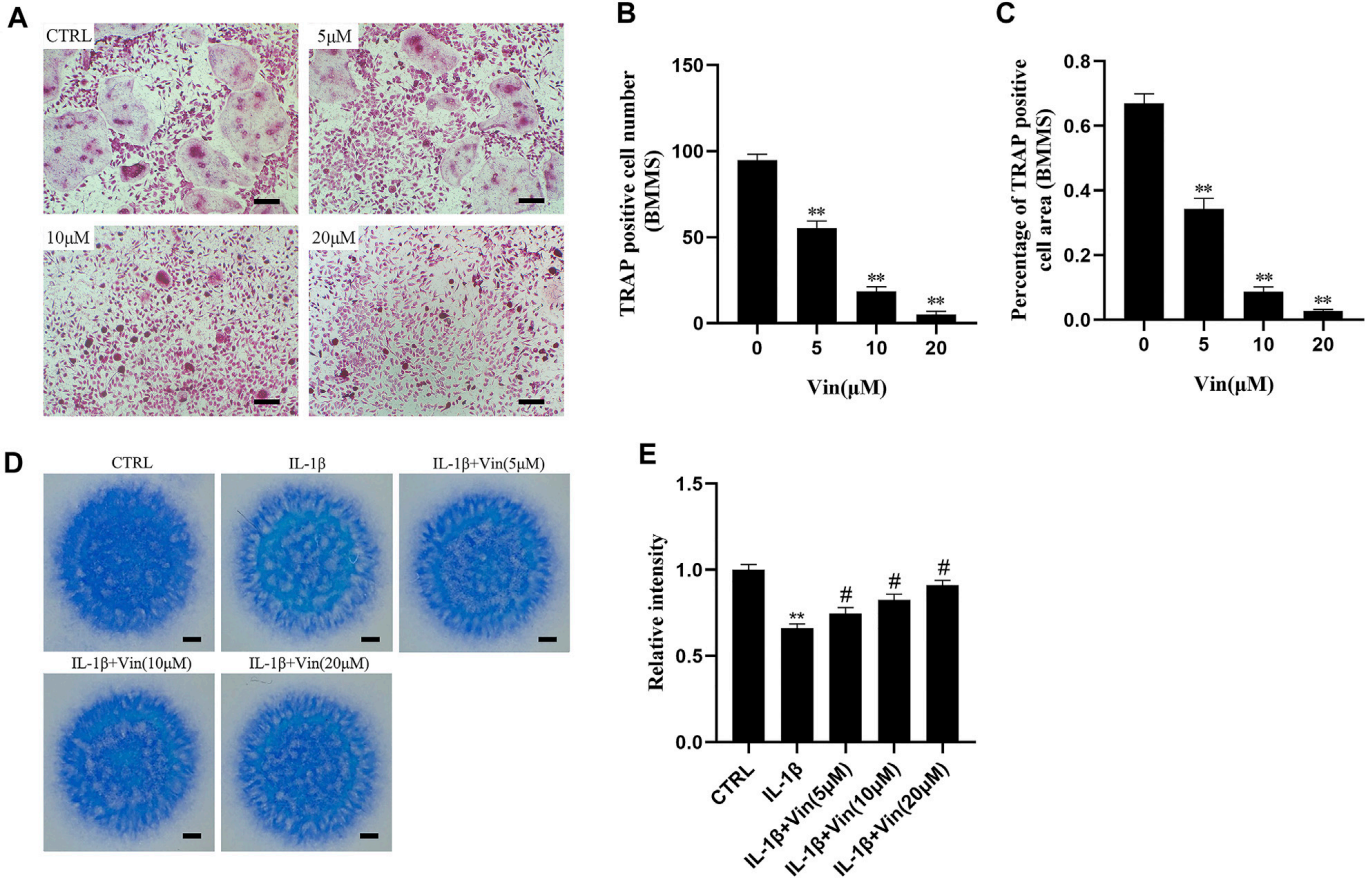
Vin inhibits the formation of osteoclasts induced by RANKL in BMMs, and stabilizes the disorders of the ECM homeostasis stimulated by IL-1β. **(A)** Tartrate-resistant acid phosphatase (TRAP) staining results for BMMs cultured with M-CSF (30 ng/ml), RANKL (50 ng/ml), and different concentrations of Vin (0, 5, 10, and 20 μM) for 5–7 days. Scale bar = 200 μm. **(B,C)** TRAP-positive multinuclear cell numbers and area. **(D)** Toluidine blue staining results for ATDC5 cultured with IL-1β (10 ng/ml) and various concentrations of Vin (0, 5, 10, and 20 μM) for 7–9 days by high density culture method. Scale bar = 200 μm. **(E)** The relative intensity of blue staining. (*p < 0.05; **p < 0.01 vs. the control group; #*p* < 0.05; ##*p* < 0.01 vs. the IL-1β-treated group).

The concentrations of 0, 5, 10, or 20 μM (Vin) were selected for the subsequent experiments. As shown in Figures 2A–C, in the control group, BMMs were induced into mature TRAP-positive multinucleated osteoclasts in the presence of M-CSF and RANKL. However, the number and the area of TRAP-positive multinucleated osteoclasts were reduced dose-dependently in Vin-treated groups. The number of osteoclasts was 55.33 ± 4.163 per well with 5 μM Vin, 18.67 ± 2.517 per well with 10 μM Vin and 5 ± 2 per well with 20 μM Vin, all of which were significantly lower than control group (*p* < 0.05).

IL-1β plays a vital part in cartilage degradation by not only downregulating the synthesis of ECM components, but also enhancing the degradation of ECM components. IL-1β has been used as a classical methodological approach to induce OA models in vitro. To examine the possible protective effects of Vin on ECM in vitro, ATDC5 cells in high density culture were stimulated with IL-1β in the absence or presence of various concentrations of Vin for 7–9 days, and toluidine blue staining was used to identify the cartilaginous matrix. (Yan et al., 2021). As shown in Figures 2D,E, the result of toluidine blue staining showed that the intensity of blue staining in the IL-1β-stimulated group was lower than that in the control group. In contrast, compared with IL-1β-stimulated group, the intensity of blue staining could be increased to 0.7467 ± 0.03512, 0.8267 ± 0.03215 and 0.9100 ± 0.03000 after treatment with 5, 10, and 20 μM Vin, respectively (*p* < 0.05). These results suggest that Vin stabilizes the disorders of the ECM homeostasis stimulated by IL-1β.

### Vin Downregulates Osteoclast-Related Gene Expressions and Chondrocytes Degeneration Gene Expressions

**FIGURE 3:**
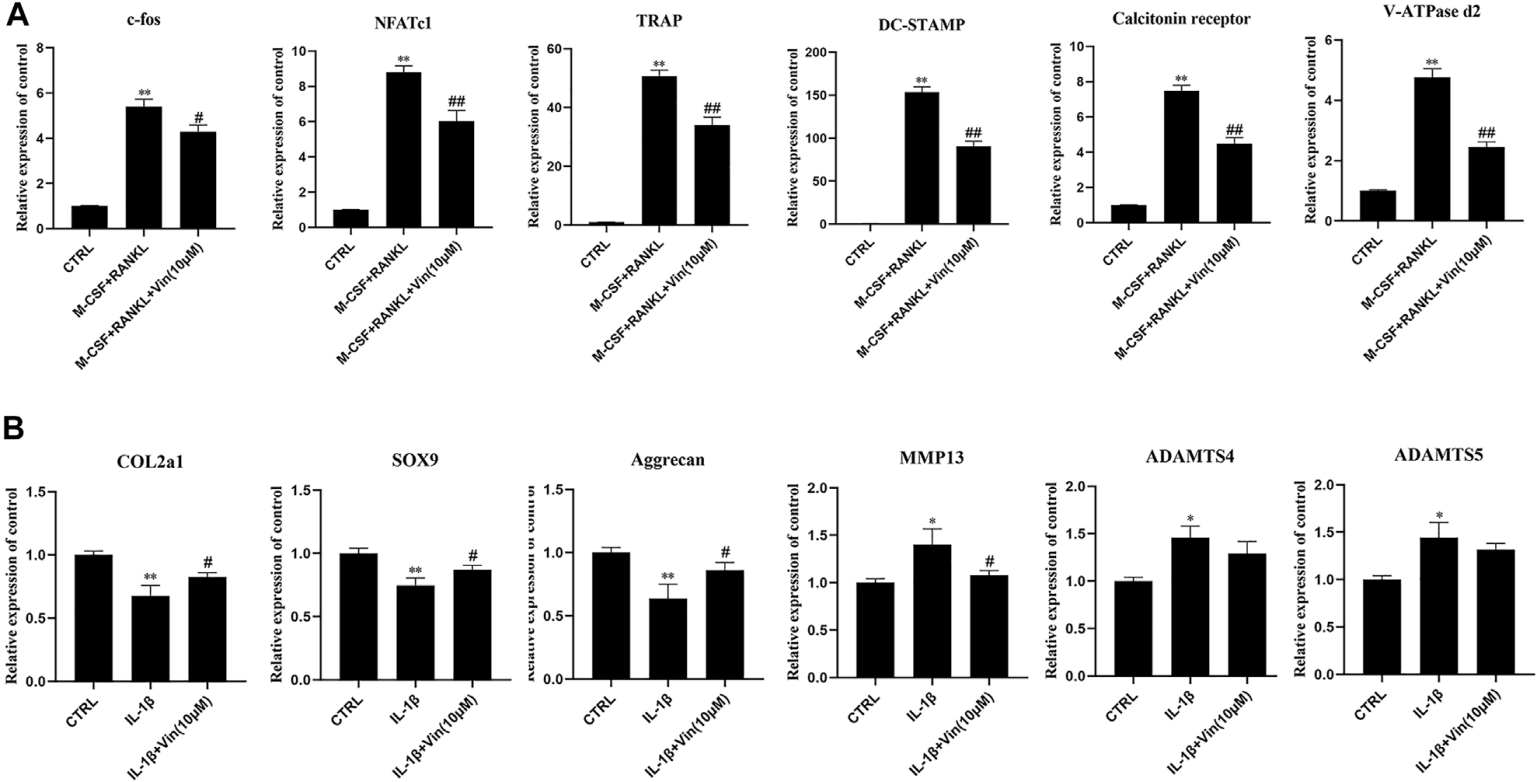
Vin suppresses osteoclast-related and IL-1β-induced gene expression. **(A)** The osteoclast-related gene expressions (c-fos, NFATc1, TRAP, DC-STAMP, calcitonin receptor, and V-ATPase d2) were analyzed using quantitative PCR. **(B)** The IL-1β-induced gene expressions (COL2a1, SOX9, aggrecan, MMP13, ADAMTS4 and ADAMTS5) were assessed by quantitative PCR. (*p < 0.05; **p < 0.01 vs the control group; #*p* < 0.05; ##*p* < 0.01 vs the M-CSF and RANKL groups or the IL-1β-treated group).

Next, we evaluated the effect of Vin on the expressions of osteoclast-related genes, including c-fos, NFATc1, TRAP, DC-STAMP, calcitonin receptor, and V-ATPase d2, which play essential roles in the process of osteoclast differentiation. The quantitative PCR results showed that compared with control group, osteoclast-specific gene expressions were increased in the presence of M-CSF and RANKL. However, these gene expressions were suppressed by Vin **(** Figure 3A). In addition, we explored the effects of Vin on COL2a1, SOX9, aggrecan, MMP13, ADAMTS4 and ADAMTS5 mRNA expression in IL-1β-induced ATDC5 cells. The quantitative PCR results revealed that IL-1β-stimulation of ATDC5 cells showed a significant upregulation of MMP13, ADAMTS4 and ADAMTS5 mRNA expressions and downregulation of COL2a1, SOX9 and aggrecan mRNA expressions in contrast with the control group. Whereas, Vin suppressed the IL-1β-induced mRNA expression of MMP13 and upregulated COL2a1, SOX9 and aggrecan mRNA expressions without affecting ADAMTS4 and ADAMTS5 gene expressions **(** Figure 3B **)**.

### Vin Suppresses the NF-κB and ERK Signaling Pathways in RAW264.7 Cells

**FIGURE 4:**
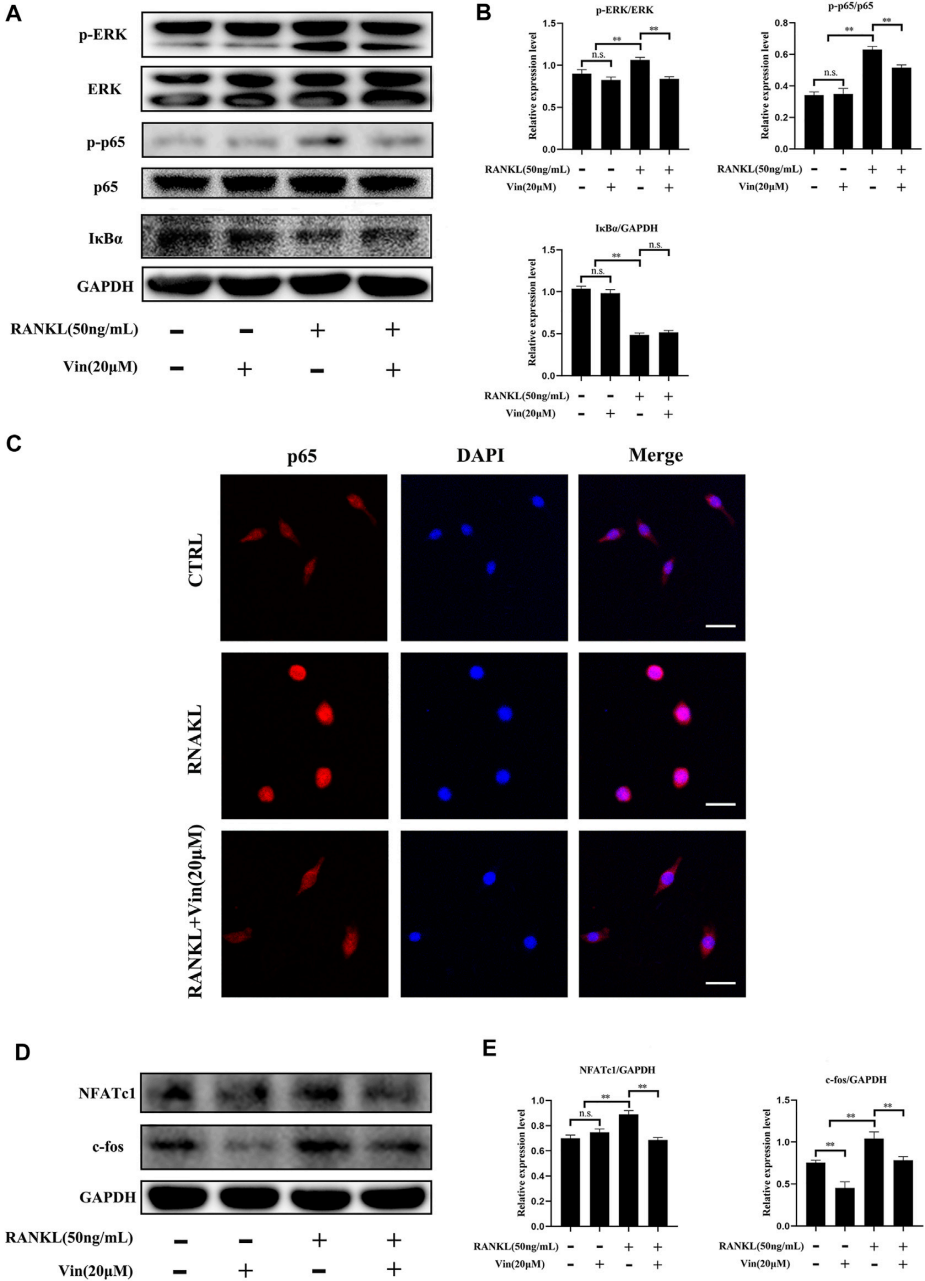
Vin suppresses the signaling pathways of NF-κB and ERK induced by RANKL, and inhibits c-fos and NFATc1 expressions induced by RANKL. **(A)** RAW264.7 cells were pretreated with or without Vin (20 μM) for 2 h followed by 0 or 50 ng/ml RANKL for 10 min. **(B)** The protein levels of p-ERK/ERK, p-p65/p65, IκBα/GAPDH were quantified by ImageJ software. **(C)** Nuclear translocation of p65 in RAW264.7 was determined using immunofluorescence. Scale bar = 10 μm. **(D)** RAW264.7 cells were pretreated with or without Vin (20 μM) for 2 h followed by 0 or 50 ng/ml RANKL for 48 h. **(E)** ImageJ was used to quantify the protein levels of NFATc1/GAPDH and c-fos/GAPDH. (*p < 0.05; **p < 0.01).

Previous studies have revealed that NF-κB and ERK play key roles in osteoclastogenesis. (Nakamura et al., 2003; Abu-Amer, 2013; Li et al., 2019). Therefore, we relied on western blot and p65 immunofluorescence staining to investigate whether these signaling pathways were involved in osteoclastogenesis suppression mediated by Vin. Western blot results showed that RANKL treatment greatly caused degradation of IκBα (an inhibitor of NF-κB), but increased phosphorylation of p65 in RAW264.7 cells. However, Vin treatment could diminish p65 phosphorylation without affecting IκBα degradation **(** Figure 4A). Meanwhile, p65 immunofluorescence staining revealed that Vin significantly suppressed the nuclear translocation of p65 **(** Figure 4C **)**. These results indicated that Vin inhibited the activation of NF-κB signaling pathway. ERK signaling pathway is another equivalently important for the osteoclast differentiation. In our study, RANKL stimulation significantly promoted the phosphorylation of ERK in RAW264.7 cells, while Vin attenuated the effect after stimulation (Figure 4A). These observations were confirmed by quantitative analysis (Figure 4B). Taken together, these data suggest that Vin inhibits osteoclastogenesis mainly *via* the NF-κB and ERK signaling pathways. NF-κB and ERK activation triggers the factors of osteoclast differentiation, such as NFATc1 and c-fos which are considered crucial regulators to initiate osteoclast differentiation. Thus, we examined the protein levels of NFATc1 and c-fos by Vin treatment. NFATc1 and c-fos were dramatically increased by RANKL treatment, but Vin treatment reduced the effect after stimulation compared with control treatment. Quantitative analysis confirmed these observations **(** Figures 4D,E **)**.

### Vin Reduces IL-1β-induced NF-κB and ERK Signaling Pathways in ATDC5 Cells

**FIGURE 5:**
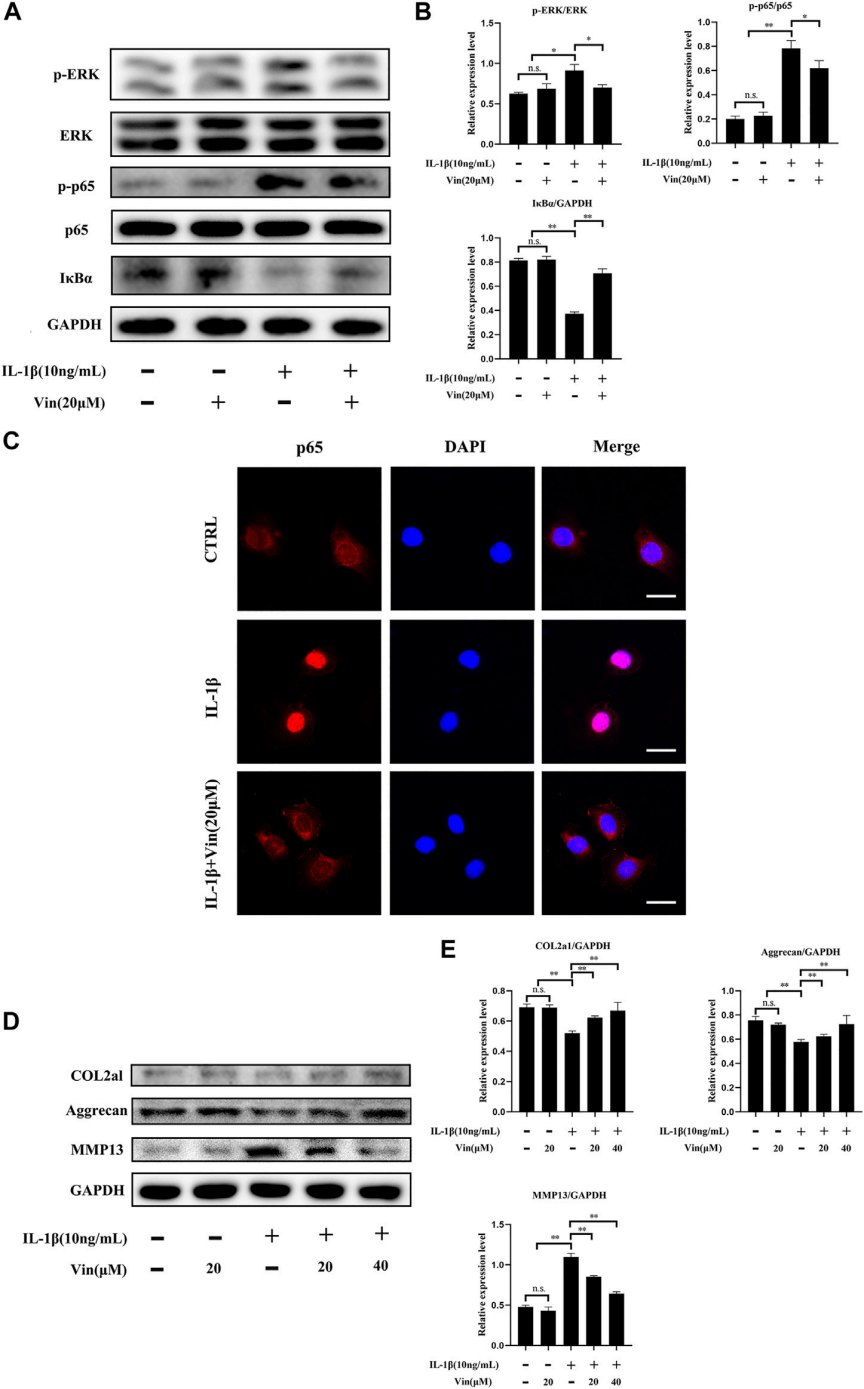
Vin suppresses NF-κB and ERK pathways stimulated by IL-1β, and inhibits the degradation of COL2a1 and aggrecan, and the expression of MMP13. **(A)** ATDC5 cells were pretreated with or without Vin (20 μM) for 2 h followed by 0 or 10 ng/ml IL-1β for 30 min. **(B)** The protein levels of p-ERK/ERK, p-p65/p65, IκBα/GAPDH were quantified by ImageJ software. **(C)** Nuclear translocation of p65 in ATDC5 was determined using immunofluorescence. Scale bar = 10 μm. **(D)** ATDC5 cells were pretreated with or without Vin (20 μM) for 2 h followed by 0 or 10 ng/ml IL-1β for 48 h. **(E)** ImageJ quantification of COL2a1/GAPDH, aggrecan/GAPDH and MMP13/GAPDH protein levels. (*p < 0.05; **p < 0.01).

NF-κB and ERK signaling pathways are crucial in cartilage degeneration. (Loeser et al., 2008; Choi et al., 2019). To uncover whether these signaling pathways were inhibited in cartilage degeneration by Vin, western blot and p65 immunofluorescence staining were conducted as aforementioned. Our results revealed that the phosphorylation of p65 and the degradation of IκBα were significantly prevented by Vin treatment in IL-1β-induced ATDC5 cells. In addition, with RANKL’s stimulation, the phosphorylation of ERK was increased and this trend was inhibited by Vin treatment after stimulation **(** Figure 5A). Quantitative analysis confirmed these observations (Figure 5B). In the same way, we performed p65 immunofluorescence staining in ATDC5 cells and found that the nuclear translocation of p65 was also suppressed by the Vin treatment (Figure 5C). Besides, we measured the protein levels of COL2a1, aggrecan and MMP13, which play important roles in ECM homeostasis, and found that Vin treatment significantly inhibited the IL-1β-stimulated expression of MMP13 and increased the expressions of COL2a1 and aggrecan (Figures 5D,E). Collectively, Vin attenuates NF-κB and ERK signaling pathways activation thereby stabilizing ECM homeostasis in cartilage.

### Vin Protects Against OA in DMM-Induced Mouse Model

**FIGURE 6:**
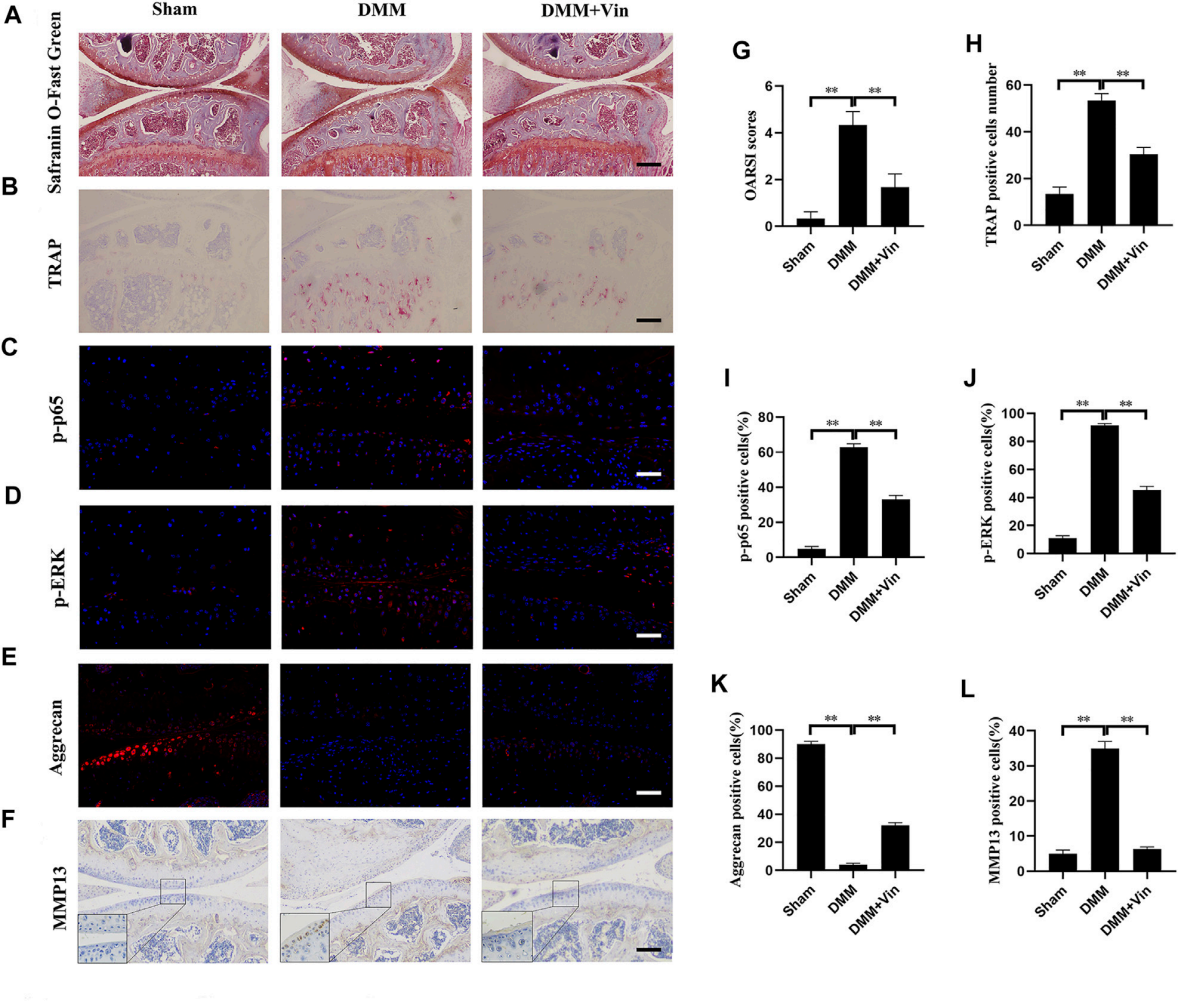
Vin protects against cartilage degeneration and osteoclast activity in a DMM-induced OA mouse model *in vivo*. **(A)** The effect of Vin on DMM-induced cartilage degeneration was observed by Safranin O-Fast Green staining. **(B)** Osteoclasts in subchondral bone were stained by TRAP **(C–F)** Immunofluorescence (p-p65, p-ERK and aggrecan) and immunohistochemical (MMP13) staining of chondrocytes in articular cartilage. **(G–L)** Quantitative analysis of OARSI scores and positively stained cells in articular cartilage. All scale bar = 200 μm. (*p < 0.05; **p < 0.01).

DMM-induced OA mouse model was established to evaluate the effects of Vin on OA *in vivo*. Knee joint samples were collected and subjected to Safranin O-Fast Green staining, TRAP staining, immunofluorescence (p-p65, p-ERK and aggrecan) and immunohistochemical (MMP13) staining. Safranin O-Fast Green staining revealed that compared with the sham group, vast proteoglycan loss, and verical clefts/erosion to the calcified cartilage were observed in the DMM group. Interestingly, Vin significantly suppressed the proteoglycan loss and cartilage destruction, as compared with the DMM group (Figure 6A). These findings were consistent with the results of OARSI scores which were increased significantly in DMM group but decreased significantly with Vin treatment (Figure 6G). Moreover, TRAP staining demonstrated that the number of TRAP-positive multinucleated cells in subchondral bone was decreased following Vin treatment (Figures 6B,H). To verify whether Vin attenuated p-p65 and p-ERK, and stabilized ECM homeostasis during OA, we performed immunofluorescence (p-p65, p-ERK and aggrecan) and immunohistochemical (MMP13) staining, and found that the expression of p-p65, p-ERK, MMP13 and the degradation of COL2a1 were inhibited by Vin treatment (Figures 6C–F,I–L). To evaluate whether Vin had systemic effects in mouse model. We performed the HE experiments of heart, liver, spleen, lung, kidney (Supplementary Material), and found that Vin treatment had no significant negative effects on the mouse organs. These results demonstrate the protective effect of Vin in DMM-induced OA by suppressing cartilage degeneration and osteoclast activity *in vivo.*

## Discussion

OA has long been considered as a degenerative disease with a high prevalence and an economical burden. (Shi et al., 2019). Although non-steroidal anti-inflammatory drugs are widely used in the treatment of OA, they only temporarily alleviate clinic symptoms and have some serious side effects. (Dona et al., 2016; Moore et al., 2019). Therefore, there is an urgent need to explore safe and effective treatments for OA. With the increased depth of research on OA, the pathogenesis of OA is associated with not only articular cartilage but also subchondral bone remodeling. (Burr and Gallant, 2012; Suri and Walsh, 2012). The crosstalk among osteochondral units is greatly important. (Goldring and Goldring, 2016). Therefore, targeting articular cartilage alone may be insufficient to prevent the progression of OA. Treatment on subchondral bone remodeling could be added and may be promising choices for OA. (Karsdal et al., 2008; Zhen and Cao, 2014; Li et al., 2021). Previous studies confirmed the beneficial effects on OA by the treatment targeting articular cartilage and subchondral bone remodeling. (Karsdal et al., 2008; Zhen and Cao, 2014; Li et al., 2021).

According to previous studies, (Goboza et al., 2019; Zhan et al., 2020), Vin could inhibit osteoclastogenesis and inflammation significantly, which enables us to hypothesize that Vin may suppress the progress of OA by suppressing osteoclastogenesis and stabilizing ECM of articular cartilage. In the present study, we demonstrated that Vin with nontoxic concentrations significantly inhibited the RANKL-induced osteoclast differentiation, which was consistent with the previous report. (Zhan et al., 2020). High density culture for ATDC5 cells demonstrated the protective effect of Vin, which stabilized the disorders of the ECM homeostasis induced by IL-1β. These results suggest that Vin could inhibit osteoclastogenesis and stabilize ECM of articular cartilage, exhibiting therapeutic potential for OA.

Then, the molecular mechanisms were further explored on Vin-mediated inhibition of osteoclast differentiation and the ECM stabilization. NF-κB and ERK signaling pathways play key roles in osteoclastogenesis, (Nakamura et al., 2003; Abu-Amer, 2013; Li et al., 2019), and OA development. (Loeser et al., 2008; Yan et al., 2012; Choi et al., 2019). In osteoclast differentiation, NF-κB and ERK signaling pathways can be activated by RANKL binding to its receptor RANK, which trigger the activation and nuclear translocation of osteoclast transcription factors (Abu-Amer, 2013; Ouyang et al., 2014). In the classical NF-κB pathway, IκBα can attach to NF-κB protein including the p65 subunit, inhibiting its phosphorylation and migration to the nucleus, where specific DNA sites are bound to activate the specific genes. (Abu-Amer, 2013). In addition, ERK induces c-fos for osteoclastogenesis. (Monje et al., 2005). RANKL-induced osteoclastogenesis could be prevented by ERK inactivation. (Ouyang et al., 2014). In the present study, Vin inhibited the activation of NF-κB in osteoclast differentiation by suppressing the phosphorylation of p65 and the nuclear translocation of p65. The ERK signaling pathway in osteoclastogenesis was also inhibited by suppressing ERK phosphorylation. Furthermore, the NF-κB and ERK signaling pathways play crucial roles in the cartilage destruction mediated by OA. (Loeser et al., 2008; Choi et al., 2019). Our results indicated that Vin specifically decreased NF-κB and ERK activation induced by IL-1β, as evidenced by reduced IκBα degradation, p65 phosphorylation and p65 nuclear translocation, and ERK phosphorylation after Vin treatment. In summary, our results suggest that Vin decreases osteoclast differentiation and cartilage destruction by inhibiting NF-κB and ERK pathways.

**FIGURE 7:**
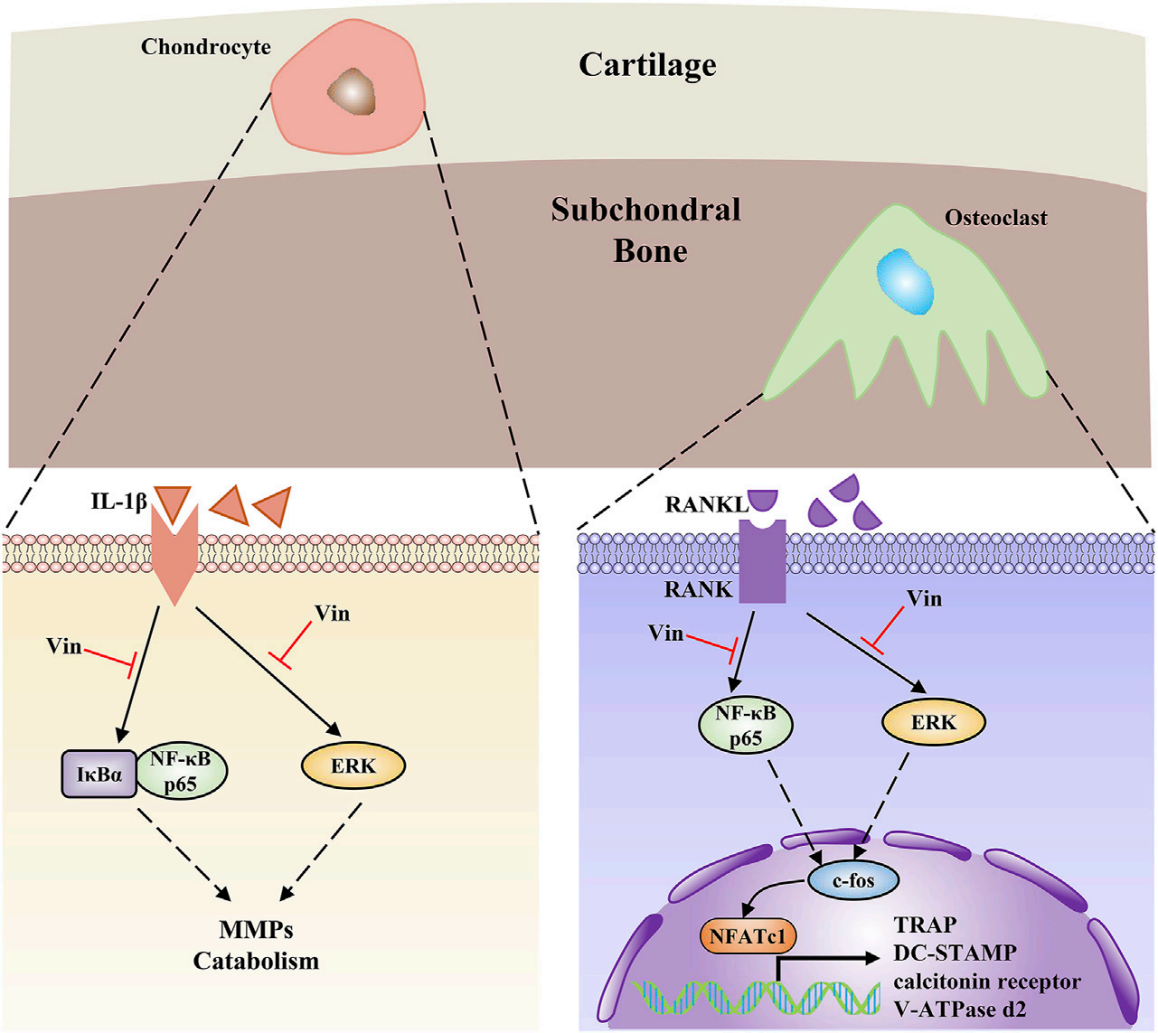
The schema elucidates Vin as a promising therapeutic to treat OA *via* suppressing the NF-κB and ERK pathways in both chondrocytes and subchondral osteoclasts.

NF-κB and ERK activation triggers the factors of osteoclasts differentiation, such as c-fos and NFATc1 (Monje et al., 2005; Abu-Amer, 2013). As crucial regulators for osteoclast differentiation and activity, c-fos and NFATc1, regulate the expressions of osteoclast-specific genes, such as TRAP, DC-STAMP, calcitonin receptor, and V-ATPase d2, all of which influence osteoclast precursor differentiation into mature osteoclasts. (Ouyang et al., 2014; Zhu et al., 2021). In this study, we found that expressions of the above genes were inhibited by Vin treatment, thereby blocking osteoclast formation. Additionally, inhibition of c-fos and NFATc1 expressions by Vin further served as evidence of the protein levels from western blot assay. In cartilage, NF-κB and ERK involved in the expressions of MMPs and other mediators involved in OA development. (Loeser et al., 2008; Choi et al., 2019). MMP13, ADAMTS4, and ADAMTS5 are key regulators of cartilage destruction. (Kapoor et al., 2011). SOX9, a master regulator of chondrogenesis, efficiently binds to single or double high-mobility group (HMG)-box site(s) in DNA and thereby transactivates its target genes, such as COL2a1 and aggrecan, which participate in ECM formation. (Song and Park, 2020). Our findings revealed that Vin specifically downregulated the mRNA expression of MMP13 and upregulated the mRNA expressions of COL2a1, aggrecan, and SOX9, but did not affect the expressions of ADAMTS4 and ADAMTS5. Western blot also revealed that the degradation of COL2a1 and aggrecan, and the expression of MMP13 were suppressed after Vin treatment (Figure 7).

Considering the effect of Vin in inhibiting osteoclastogenesis and stabilizing ECM *in vitro,* we further examined its effect on a DMM-induced OA mouse model, which is similar to human OA and has been widely used to evaluate the effectiveness of hypothesized therapeutic drugs. (Glasson et al., 2007). Vin treatment could suppress the proteoglycan loss and cartilage destruction in OA mice. Vin treatment also reduced the number of TRAP-positive cells in the subchondral bone of OA mice, indicating that Vin suppresses osteoclast formation to decrease subchondral bone remodeling. Furthermore, the expression of p-p65, p-ERK, MMP13 and the degradation of aggrecan in cartilage, which led to the ECM stabilization and the inhibition of cartilage degeneration, were suppressed by Vin treatment. These results collectively suggested that Vin inhibited osteoclast formation and stabilized ECM of cartilage in OA mice by suppressing NF-κB and ERK pathways, which is consist with our *in vitro* results.

This study had certain limitations. Firstly, owing to the importance of NF-κB/ERK in osteoclastogenesis and OA, we just evaluated the effects of Vin on the two signaling pathways. It remains unknown whether Vin can affect other mediators and signaling pathways involved in osteoclastogenesis and OA. In addition, only the pharmacological effects of Vin were analyzed in this study. It involves many aspects before developing Vin into a therapeutic drug for OA, such as typical ADME characteristics including oral bioavailability, drug-likeness and so on.

## Conclusion

Our findings show that Vin inhibits osteoclastogenesis and stabilizes ECM of cartilage by suppressing the NF-κB and ERK signaling cascades. Furthermore, the dual effects of Vin *in vivo* were confirmed with DMM-induced OA mouse model. We can therefore conclude that Vin has significant potential for the treatment of OA and should be explored in future studies.

## Data Availability

The original contributions presented in the study are included in the article/Supplementary Material. Further inquiries can be directed to the corresponding authors.

## Glossary

ADAMTS4: a disintegrin and metalloproteinase with thrombospondin motifs 4
BMMs: bone marrow macrophage cells
COL2a1: type II collagen
CCK-8: cell counting kit-8
DMEM: dulbecco’s modified Eagle’s medium
DC-STAMP: dendritic cell-specific transmembrane protein
DMM: destabilization of the medial meniscus
ECM: extracellular matrix
EDTA: ethylenediaminetetraacetic acid
ERK: extracellular signal–regulated kinase
FBS: fetal bovine serum
GAPDH: glyceraldehyde-3-phosphate dehydrogenase
IL-1β: interleukin-1 beta
IκBα: inhibitor of NF-κB
MMPs: matrix metalloproteinases
M-CSF: macrophage colony-stimulating factor
NFATc1: nuclear factor of activated T cells 1
NF-κB: receptor activator of nuclear factor kappa B
OA: osteoarthritis
OARSI: Osteoarthritis Research Society International
PBS: phosphate-buffered saline
qPCR: quantitative real-time polymerase chain reaction
RANKL: receptor activator of nuclear factor kappa B ligand
RIPA: radioimmunoprecipitation assay
SOX9: sex-determining region Y box 9
TRAP: tartrate-resistant acid phosphatase
Vin: Vindoline
V-ATPase d2: vacuolar-type ATPase d2
