# Supplementary figures and images for: Vindoline Attenuates Osteoarthritis Progression Through Suppressing the NF-κB and ERK Pathways in Both Chondrocytes and Subchondral Osteoclasts
Source: Front Pharmacol. 2022 Jan 12;12:764598. doi: 10.3389/fphar.2021.764598 (PMC8790248; doi:10.3389/fphar.2021.764598)

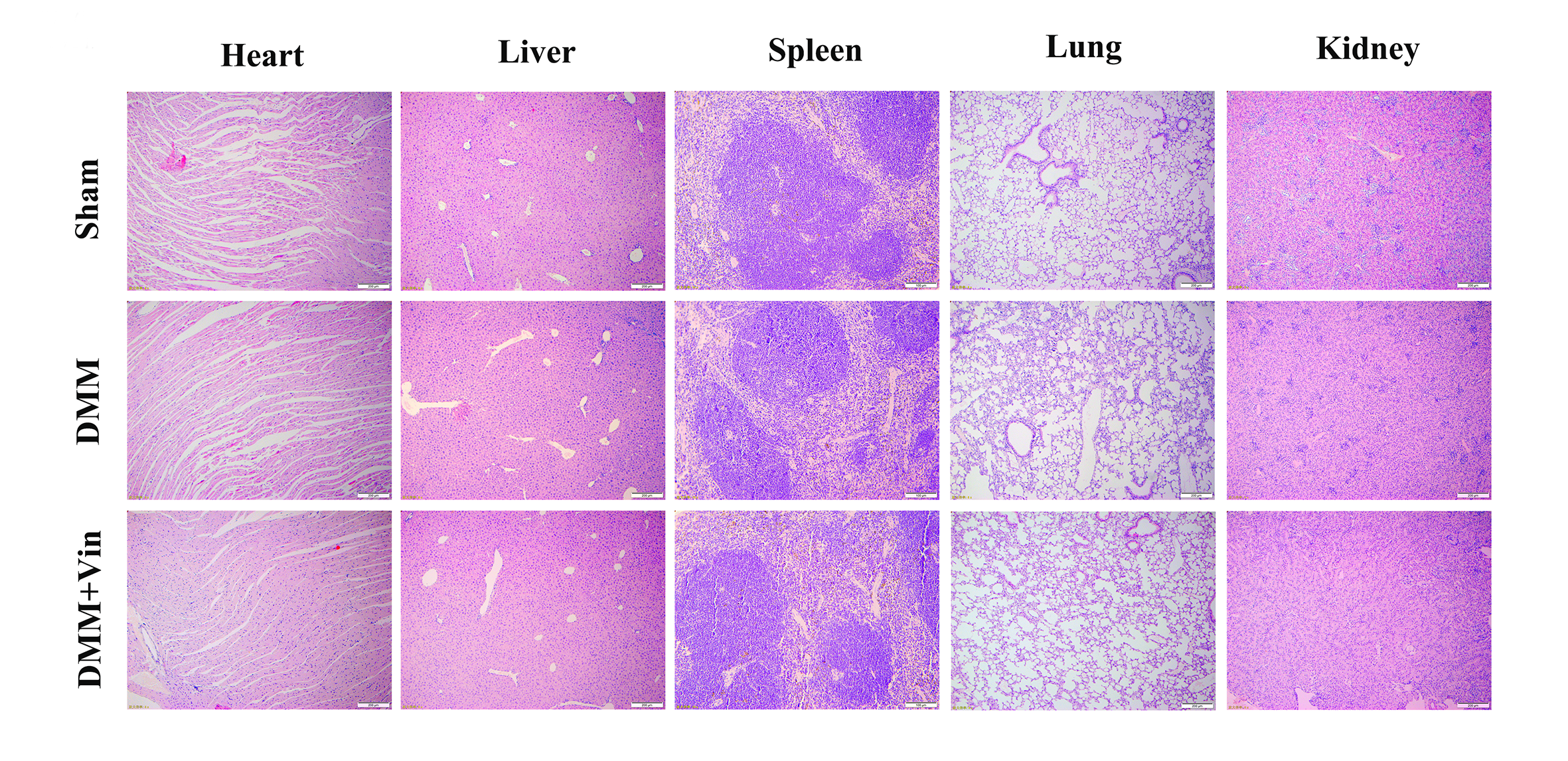

Supplement: Supplementary file 1 [file Image1.TIF]
